# Metabolic syndrome in overweight children from the city of Botucatu - São Paulo State - Brazil: agreement among six diagnostic criteria

**DOI:** 10.1186/1758-5996-2-39

**Published:** 2010-06-09

**Authors:** Ana Elisa M Rinaldi, Gustavo D Pimentel, Avany F Pereira, Gleice FCP Gabriel, Fernando Moreto, Roberto C Burini

**Affiliations:** 1 Uberlândia School of Medicine, Nutrition Course, Uberlândia Federal University - UFU, Uberlândia, MG, Brazil; 2 Exercise and Nutrition Metabolism Center (CeMENutri), Botucatu School of Medicine - UNESP, Botucatu, SP, Brazil; 3 Department of Physiology, Nutrition Physiology Division - UNIFESP, São Paulo, SP, Brazil; 4 Department of Nutrition and Dietetics - Rio de Janeiro Federal University - UFRJ, Rio de Janeiro, RJ, Brazil; 5 Department of Internal Medicine, Course of Physiopathology in Internal Medicine, Botucatu School of Medicine - UNESP, Botucatu, SP, Brazil; 6 Department of Pathology, Botucatu School of Medicine - UNESP, Botucatu, SP, Brazil; 7 Department of Public Health, Exercise and Nutrition Metabolism Center (CeMENutri), Botucatu School of Medicine - UNESP, Botucatu, SP, Brazil

## Abstract

**Background:**

The metabolic syndrome has been described in children; however, a standard criterion has not been established for its diagnosis. Also, few studies have been conducted to specifically observe the possible existence of agreement among the existing diagnostic criteria. The purpose of the study is to evaluate agreement concerning prevalence rates of the metabolic syndrome diagnosed by six different criteria in overweight schoolchildren in the city of Botucatu - SP -Brazil.

**Methods:**

This is a cross-sectional study on 128 overweight schoolchildren. Clinical examination included anthropometry, pubertal staging evaluation, and blood pressure. Triacylglycerol, glycemia, HDL-cholesterol, insulin levels, and HOMA-IR were determined. The Kappa index, the Mann-Whitney test and the chi-square test were used for statistical analysis.

**Results:**

The prevalence of the metabolic syndrome varied from 10 to 16.5% according to different diagnostic criteria. Results were similar for boys and girls and pubertal stage. Great agreement was observed among the six different diagnostic criteria for the metabolic syndrome.

**Conclusions:**

Different diagnostic criteria, when adopted for subjects with similar demographic characteristics, generate similar and compatible prevalence. Results suggest that it is possible to adopt any of the analyzed criteria, and the choice should be according to the components available for each situation.

## Background

Insulin resistance was firstly described by Reaven (1988) [1] as the concomitant presence of abdominal adiposity, dyslipidemia, hypertension and insulin resistance or type-2 diabetes mellitus, and it is regarded as the main risk factor for cardiovascular diseases.

The term syndrome was firstly used to describe a set of risk factors utilized by a group of researchers whose main focus was diabetes mellitus [2]. Reaven (1988) [1] described syndrome X as a cluster of factors such as abdominal adiposity, dyslipidemia, hypertension and insulin resistance, emphasizing the role played by the latter as a possible precursor factor for the other diseases. That syndrome was called by other researchers as the insulin resistance syndrome, concentrating the discussion on diabetes mellitus and hyperinsulinemia as possible causal factors and disregarding overweight [3,4]. Later, the Adult Treatment Panel III -National Cholesterol Education Program (ATPIII-NCEP) began to refer to that syndrome as the metabolic syndrome, with the purpose to use the term in the more global context and consider other causal factors related with abdominal fat excess [5].

Several studies [6-8] have evaluated the prevalence of the metabolic syndrome in children or the isolated presence of its components, despite the inexistence of a single diagnostic criterion. Disagreement concerning the choice for the best diagnostic criterion mainly results from the difficulty in establishing cutoff points due to the absence of clinical manifestations of cardiovascular diseases in childhood [9]. There are doubts whether cutoff points must be absolute or expressed in percentiles considering age, gender and pubertal stage due to rapid growth in childhood and mainly in adolescence [10].

As regards the prevalence of the metabolic syndrome at this age range, data disagree according to the region, diagnostic criteria and cutoff points adopted. In a population-based study, the prevalence of the metabolic syndrome in American adolescents was 4.2% for the general sample and 28.7% among overweight individuals [8]. Cruz et al. (2004) [11] found a 30% rate for the metabolic syndrome in obese children and adolescents by adopting another similar criterion to that previously cited; however, that was achieved by fixing the cutoff points in percentiles according to age and gender.

In Spain [12], the prevalence of the metabolic syndrome was 18% among obese children and adolescents. It was a similar result to that observed in a French study, which found 15.9% [13]. Nevertheless, in the National Chinese Study on Health and Nutrition with a representative sample of children and adolescents, prevalence was 1.5% in eutrophic, 18.3% in overweight and 38.1% in obese subjects [14].

In Brazil, data concerning the metabolic syndrome in childhood are available from regional studies. Those including overweight children and adolescents observed a prevalence rate for the metabolic syndrome from 4 to 42.4% [15-18]. The diagnostic criteria used in the studies were those proposed by the National Cholesterol Education Program (NCEP) [5] and WHO (1998) [19] with adaptations for age and cutoff points.

Despite the large number of studies available on the prevalence of the metabolic syndrome in the pediatric population, few have been conducted to specifically observe the possible existence of agreement among diagnostic criteria, and studies of this type only show its variation when different criteria are adopted [20,21].

This study aimed at analyzing agreement concerning the prevalence of the metabolic syndrome as diagnosed by six different criteria in overweight schoolchildren in the city of Botucatu, São Paulo State - Brazil.

## Methods

This is a cross-sectional study conducted in three primary schools (private, public and non-governmental) in Botucatu/SP - Brazil from June to December 2007. The schools were selected based on approval by their principals and from the city's Secretary of Education.

The study was approved by the Research Ethics Committee of the Botucatu School of Medicine - UNESP, according to official letter number 579/2006, as well as by that of the Faculty of Pharmaceutical Sciences - USP, according to document number 78/2007. All persons responsible for the schools and the schoolchildren signed a free consent form designed in accordance with resolution no. 196/96 concerning "Studies involving human beings, by the Health Council of the Ministry of Health".

Initially, anthropometric and blood pressure measurements were performed on all 6-to-10-year-old schoolchildren (670 children) enrolled in the three schools. Later, those diagnosed as overweight (231 children), as defined by a body mass index (BMI) equal to or higher than the 85^th ^percentile, based on the reference curves developed by the National Center for Health Statistics - Center for Disease Control and Prevention (CDC, 2000) [22], were selected.

The nutritional diagnosis of overweight (BMI equal to or higher than the 85^th ^percentile and lower than the 95^th ^percentile) and/or obesity (BMI equal to or higher than the 95^th ^percentile) according to age and gender (CDC, 2000) [22] and the performance of the biochemical tests proposed were considered as criteria for inclusion in the study. The overweight schoolchildren with a clinical history of other morbidities and undergoing medical treatment and clinical-nutritional follow-up for body weight reduction were excluded.

All the children with an overweight/obesity diagnosis were contacted by telephone in order to participate in the study. Sample size estimation was based on the prevalence of overweight and obesity (18%) of this sample by adopting a 7% margin of error. Therefore, the minimum number of subjects was 115 children.

Body weight was measured by an electronic scale (Filizola^®^) to the nearest 0.1 kg while the schoolchildren were barefoot and wearing light clothes. Height was determined by a portable Seca^® ^stadiometer to the nearest 01.cm, according to norms proposed by the World Health Organization (WHO, 1995) [23]. BMI (weight in kilograms divided by the squared height in meters) was calculated by using the measured height and weight and converted to percentiles for age in months and gender by using the Center for Disease Control and Prevention (CDC, 2000) [22] growth charts and computer software Epi-Info^® ^version 3.2 (2004). Waist circumference (WC) was measured midway between the rib cage and the superior border of the iliac crest by using a millimetric non-extensible and non-elastic measuring tape (Sanny^®^) after complete expiration.

The triceps (TSF) and subscapular (SSSF) skinfold thicknesses were measured three times to the nearest 1.0 mm on the right side of the body by Lange^® ^Skinfold calipers (Cambridge Scientific Industries, Inc, Cambridge, MD), and the mean values were used in the analyses. The triceps skinfold was then measured parallel to the long axis of the arm midway between the acromion and the olecranon, with the arm slightly flexed, and the subscapular skinfold was measured below the lower angle of the left scapula at a diagonal in the natural cleavage of the skin. Fat mass was calculated from skinfold thickness measurements by using age and gender-specific equations proposed by Slaughter et al. (1988) [24]. The reference values adopted for body fat percentage were proposed by Lohman (1987) [25], and values equal to or higher than 25% for girls and equal to or higher than 20% for boys were classified as mildly high.

Blood pressure was measured by the auscultatory method after the child had been sitting at rest for a minimum period of 5 minutes, and the cuff involved 80% of the right arm's circumference. The arm rested on a support surface at the level of the precordium. Three variable cuff sizes were used according to the child's brachial circumference. Blood pressure was measured three times in three different days only when the first measurement was above 95th percentile according to gender, age and height, based on The Fourth Report on the Diagnosis, Evaluation, and Treatment of High Blood Pressure in Children and Adolescents [26].

Fasting glucose, HDL-cholesterol and triacylglycerol concentrations were quantified by using a semi-automatic spectrophotometer (Labquest^®^, Labtest Diagnóstica) and commercial kits (Labtest Diagnóstica) by the enzymatic colorimetry method. The serum aliquots were stored at a temperature of -80°C for later insulin analysis by the immunochemical luminescence method using commercial kits (DPC Medlab) and an automated device (Immulite 2000^®^; DPC Medlab). HOMA-IR (Homeostasis Model Assessment-Insulin Resistance) was calculated according to the formula: fast insulin (UI/L) x fast glycemia (mmol/L)/22.5, as proposed by Levy et al (1998) [27].

Pubertal stage was assessed in all children by a pediatrician using the 5-stage scale for breast development and pubic hair in girls; testicular size and pubic hair in boys, according to Tanner (1962) [28]. The children were thereafter categorized into 3 groups: prepubertal (Tanner stage 1), pubertal (Tanner stage 2-4) and postpubertal (Tanner stage 5).

These criteria were selected because their components could be easily obtained and because they presented cutoff points that were already adapted to the pediatric age range. For each selected criterion, the reference values established by their respective authors (Table 1) were respected. The metabolic syndrome diagnosis was established, in all criteria, by the presence of three or more altered components.

**Table 1:** Description of the metabolic syndrome criteria and their respective cutoff points.

Metabolic syndromecriteria						
**Age group of children in the studies**	**7-11 years**	**10-19 years**	**6-11 years**	**8-12 years**	**8-16 years**	**12-19 years**

**Metabolic syndrome components**	**Ferreira et al**^**15**^	**Silva et al**^**16**^	**Boney et at**^**30**^	**Braunschweig et al**^**7**^	**Monzavi et al**^**31**^	**Cook et al**^**8**^
Anthropometric indicators*	BMI≥95^th ^percentile	BMI≥97^th ^percentile	IMC≥85^th ^percentile	WC≥90^th ^percentile	BMI≥95^th ^percentile	WC≥90^th ^percentil
Insulin resistance indicators**	Glycemia>100	HOMA>2.5	Glycemia >110	Glycemia≥110	Glycemia≥100	Glycemia≥100
HDL-C (mg/dL)	≤38	≤35	<5^th ^percentile	≤40	≤10^th ^percentile	≤40
Triglycerides (mg/dL)	≥110	≥130	>95^th ^percentile	≥110	≥90^th ^percentile	≥110
SBP/DBP (mmHg)	>95^th ^percentile	≥95^th ^percentile	>95^th ^percentile	≥90^th ^percentile	≥90^th ^percentile	≥90^th ^percentile

*BMI = body mass index or WC = waist circumference **impaired fasting glucose/HOMA-IR

After of the evaluations, the children's parents received the results (anthropometric assessment, biochemical tests and blood pressure). All the children were given nutritional advice, and those with a diagnosis for high blood pressure were referred to the pediatric nephrology outpatient unit of the Botucatu School of Medicine, Brazil.

The data were processed in software SPSS version 12.0 (SPSS Inc., Chicago, IL, USA). Data normality was evaluated by the Shapiro-Wilk Test. Descriptive statistics are displayed as arithmetic means and standard deviation. Comparison between genders for the anthropometric and biochemical data was performed by the Mann-Whitney Test. Agreement among the six different criteria was evaluated by the Kappa Index, which expresses the consistency or agreement of results when measurement or examination is repeated in identical conditions. The interpretation of results was based on the following intervals: smaller than zero, poor; from 0.00 to 0.20, little; from 0.21 to 0.40, reasonable; from 0.41 to 0.60, moderate; from 0.61 to 0.80, substantial and from 0.80 to 1.00, almost perfect [29]. The Wilcoxon test was used to compare the number of altered components in each criterion. The possible influence of gender, pubertal staging, nutritional status diagnosis by BMI (overweight and obesity) and body fat percent of the metabolic syndrome was tested by the Chi-square or Fischer's exact test. P values 0.05 were considered statistically significant.

## Results

After the schoolchildren's (N = 670) anthropometric assessment, overweight prevalence was observed in 33% of the sample (16.1% overweight and 17.7% obesity), with a proportion of obese/overweight to eutrophic individuals of 1:3. Of the overweight schoolchildren participating in the present study (n = 128), 44% (n = 56) showed overweight, and 56% (n = 72) showed obesity, with a proportion of obese/overweight individuals of 1:1. Boys showed greater alterations in BMI (percentile), mean glycemia levels and systolic pressure (Table 2). As regards pubertal stage, 67% of the schoolchildren were classified as prepubertal, 33% pubertal and none as postpubertal (data not shown).

**Table 2:** Anthropometric and biochemical characteristics of school children according to gender. Botucatu-SP, 2007.

Variables	General	Boys	Girls
	
	n = 128	n = 58	n = 70
Age (years)	8.1 ± 1.4	8.1 ± 1.4	8.1 ± 1.4
Height (m)	1.3 ± 0.1	1.3 ± 0.1	1.3 ± 0.1
Weight (kg)	41.3 ± 11.1	42.9 ± 12.3	40.0 ± 10.0
BMI (kg/m 2)	22.2 ± 3.2	22.6 ± 3.6	21.8 ± 2.7
BMI (percentile)	94.3 ± 4.3	95.3 ± 3.9^a^	93.4 ± 4.5^b^
WC (cm)	72.1 ± 9.8	74.0 ± 11.1^a^	70.6 ± 8.4^b^
TSF (mm)	20.3 ± 4.7	20.2 ± 4.8	20.4 ± 4.7
SSF (mm)	14.7 ± 5.8	14.6 ± 6.3	14.8 ± 5.3
%BF	28.9 ± 6.5	29.5 ± 7.5	28.4 ± 5.6
Glycemia (mg/dL)	83.7 ± 6.5	85.6 ± 5.9^a^	82.1 ± 6.6^b^
HDL-C (mg/dL)	47.8 ± 10.9	47.5 ± 9.7	48.0 ± 11.9
Insulin (μU/mL)	7.6 ± 5.6	7.0 ± 4.7	8.2 ± 6.2
HOMA-IR	1.6 ± 1.2	1.5 ± 1.0	1.6 ± 1.3
TG (mg/dL)	98.0 ± 51.4	99.7 ± 56.6	96.5 ± 47.0
SBP (mmHg)	98.9 ± 12.1	101.3 ± 13.6	96.9 ± 10.5
DBP (mmHg)	63.9 ± 8.4	64.9 ± 9.4	63.2 ± 7.5

Different letters indicate significant difference between genders (p < 0.05). BMI: body mass index; WC: waist circumference; TSF: triceps skinfold, SSF: subscapular skinfold, %BF: percentage body fat; HOMA-IR: Homeostasis Model Assessment-Insulin Resistance; TG: triglycerides; SBP: systolic blood pressure; DBP: diastolic blood pressure.

**Figure 1:**
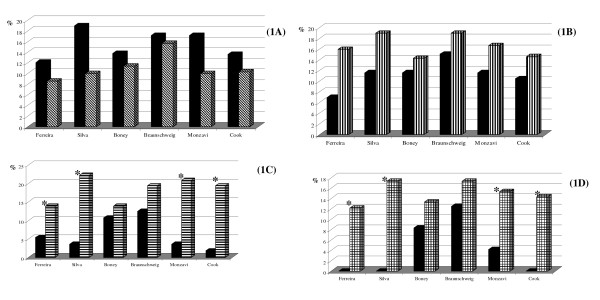
**Prevalence of Metabolic Syndrome in the six diagnosis criteria according to gender (1A), sexual maturity (1B), and nutritional diagnosis by body mass index (1C) and body fat percentage (1D) in overweight children. Botucatu-SP, 2007**. 1A -"black square" boys; "striped square" girls. 1B - "black square" prepubertal; "vertical line" pubertal. 1C - "black square" overweight; "horizontal line" obesity. 1D - "black square" normal %body fat; "checked square" elevated %body fat *p < 0.05.

The prevalence of the metabolic syndrome varied from 10 to 16.5%. The percent of altered components was more frequent in obese children according to the criteria proposed by Ferreira et al (2007) [15], Silva et al (2005) [16], Monzavi et al (2006) [30] and Cook et al (2003) [8] (p < 0.05) and in individuals with high body fat percents according to the criteria proposed by Ferreira et al (2007) [15], Silva et al (2005) [16], Monzavi et al (2006) [31] and Cook et al (2003) [8]; however, there was no influence from gender or pubertal staging (Figure 1).

Anthropometric indicators (BMI and WC), triacylglycerols, HDL-C, HOMA-IR, blood pressure and glycemia constituted the decreasing sequence of prevalences of altered components in the criteria adopted (Table 3).

**Table 3:** Percentage of overweight children with altered metabolic syndrome components according to the six diagnosis criteria.

	Diagnostic criteria					
**Altered metabolic syndrome components**	**Ferreira et al**^**15**^	**Silva et al**^**16**^	**Boney et al**^**30**^	**Braunschweig et al**^**26**^	**Monzavi et al**^**31**^	**Cook et al**^**8**^

Adiposity indicators*	57.8 (74)***	40.6 (52)	100.0 (128)	96.1 (123)	57.8 (74)	45.3 (58)
Insulin resistance indicators**	0.8 (1)	18.0 (23)	0.0 (0)	0.0 (0)	0.8 (1)	0.8 (1)
HDL-C	19.5 (25)	10.9 (14)	15.6 (20)	25.0 (32)	27.3 (35)	25.0 (32)
Triacylglycerol	26.8 (34)	16.4 (21)	29.7 (38)	26.6 (34)	30.5 (39)	26.6 (34)
SBP/DBP	11.8 (15)	12.5 (16)	11.8 (15)	14.8 (19)	14.8 (19)	14.8 (19)
Metabolic syndrome (%)	11.7 (15)	14.1 (18)	12.5 (16)	16.4 (21)	13.3 (17)	11.5 (15)

*BMI: body mass index/Waist circumference; **Fast glycemia/HOMA-IR; ***The number in all parenthesis represents the number of the children with altered metabolic syndrome components SBP/DBP (sistolic blood pressure or diastolic blood pressure).

The percent of zero or one of the syndrome's altered components was significantly higher among overweight children while the percent of two or more altered components was higher in obese children (p < 0.05) for all criteria. No children showed altered fast glycemia according to the criteria proposed by Boney et al (2005) [31] and Braunschneig et al (2005) [7], and only one showed abnormal values according to the criteria proposed by Cook et al (2003) [8], Monzavi et al (2006) [30] and Ferreira et al (2007) [15].

For all criteria, the cutoff points for blood pressure, HDL-C and triacylglycerols were similar, a fact that explains the absence of significant difference between the prevalence of alteration in these components according to the six criteria analyzed. Based on this information, it can be assumed that the differences in the prevalence of the metabolic syndrome found among the different criteria were mainly due to anthropometric indicators (BMI or WC) and to the glucose metabolism (HOMA-IR or fasting glycemia) (Table 3).

All the criteria agreed among each other, and the Kappa indexes generated values classified as moderate to almost perfect. Perfect agreement was observed among the criteria proposed by Cook et al (2003) [8] vs. Braunschneig et al (2005) [7] and Cook et al (2003) [8] vs. Ferreira et al (2007) [15]. Such result can be explained by the fact that the three criteria have been adapted from that proposed by NCEP-ATPIII with different cutoff-point adaptations. The criteria showing the least agreement among each other were those proposed by Silva et al (2005) [16] vs. Boney et al (2005) [31], with a Kappa index of 0.53, considered to be moderate agreement (Table 4).

**Table 4:** Agreement (Kappa index) among de metabolic syndrome prevalences according to six different diagnostic criteria.

*Criteria*	*Kappa Index*	*p*
Ferreira × Silva	0.67	< 0.001
Ferreira × Boney	0.73	< 0.001
Ferreira × Braunschweig	0.73	< 0.001
Ferreira × Monzavi	0.70	< 0.001
Ferreira × Cook	0.84*	< 0.001
Silva × Boney	0.53	< 0.001
Silva × Braunschweig	0.61	< 0.001
Silva × Monzavi	0.64	< 0.001
Silva × Cook	0.69	< 0.001
Boney × Braunschweig	0.78	< 0.001
Boney × Monzavi	0.62	< 0.001
Boney × Cook	0.67	< 0.001
Braunschweig × Monzavi	0.63	< 0.001
Braunschweig × Cook	0.81*	< 0.001
Monzavi × Cook	0.71	< 0.001

*perfect concordance

In these subjects, the prevalence of the metabolic syndrome, according to different criteria, showed good agreement. In the same group of subjects, the percent of children diagnosed with the metabolic syndrome seems to show little variance in function of the diagnostic criterion adopted.

## Discussion

Divergent metabolic-syndrome diagnoses at the pediatric age range encouraged the development of this Brazilian study, which is one of the first to evaluate its prevalence agreement according to different diagnostic criteria. The prevalence of the metabolic syndrome varied according to the criteria adopted; however, moderate to perfect agreement was observed among all criteria. Such criteria [7,8,15,16,30,31] were selected because the components used were easy to obtain and because they were applied to a similar age range to that in this study, except for the criterion proposed by Silva et al (2005) [16] and Cook et al (2003) [8]. The latter was selected because recent literature reviews [32,33] have shown it to be the most frequently used criterion, regardless of age range. This is certainly due to the fact that it is based on NCEP-ATPIII, which is already well founded among adults. Most of the articles available in the literature include adolescents or children in late childhood [8,34-36]. The other criteria, Silva et al (2005) [16], were selected because this study included Brazilian adolescents.

Although the six diagnostic criteria used differed in relation to the cutoff points for each component, there is agreement with regard to including indicative parameters for glycemic (fasting glycemia and HOMA-IR) and lipid (triacylglycerols, HDL-C) abnormalities, one anthropometric indicator (BMI or WC) and blood pressure. In this study, it can be observed that the components mostly contributing to prevalence variation in the metabolic syndrome were the inclusion of BMI or WC and the cutoff point adopted for each of them as well as the choice for glycemia or HOMA-IR. The other components use very similar cutoff points. Such findings are in agreement with the analysis by Brambilla et al (2007) [37] in a review study showing different prevalences according to several diagnostic criteria.

As previously mentioned, the prevalence of the metabolic syndrome in this study varied from 10 to 16.5%, without differences between gender and pubertal staging, as shown by other studies [7,11,18,20,30,35,38]. In this investigation, the anthropometric variable was (WC or BMI) the component with the highest percent of alteration, a result which is in accordance with findings in other studies [7,15,31].

As in other investigations, the present study also showed the inexistence, among children at this age range, of altered fasting glycemia. However, Golley et al (2006) [21] point out the importance of analyzing fasting insulinemia, since the number of components of the metabolic syndrome and their prevalence significantly increased according to fasting insulinemia quartiles. Ferreira et al (2007) [15] reported that the higher HOMA-IR tercile, the higher the risk factors for cardiovascular diseases. A Brazilian study conducted on overweight prepubertal children reported that a higher HOMS-IR than 2.5 showed good sensitivity and specificity to detect the metabolic syndrome [38].

Another aspect pointed out in some studies is the need to adapt cutoff points according to age [20,21] Reinehr et al (2007) [17] compared four diagnostic criteria for adults and four for children/adolescents. When using the criteria for adults, prevalence of the metabolic syndrome varied from 6 to 14%, and among the criteria for children/adolescents, it ranged from 18 to 39%. Golley et al (2006) [21] showed that prevalence in 6-to-9-year-old children according to two criteria for adults was 4%; however, when the same criteria were adjusted according to that age range, prevalence was 39%. The authors also point out that the criteria including total cholesterol and triglycerides and WC can explain most of the prevalence variation in the metabolic syndrome.

The prevalence of the metabolic syndrome found in the present study was similar to that in studies adopted as reference for this investigation, except for the criterion by Monzavi et al (2006) [30], which identified, in their population, a prevalence of 49%. By that criterion, the prevalence of the metabolic syndrome found was 13%, and it can be explained by the higher percent of children with severe obesity in those authors' sample [30].

Agreement among the different criteria varied from moderate to almost perfect. This result was expected since the criteria proposed by Ferreira et al (2007) [15], Braunschneig et al (2005) [7] and Cook et al (2003) [8] are adapted from NCEP-ATPIII for adults; however, with different cutoff points. Seo et al (2008) [36], in a similar study to the present investigation, found great agreement (88.7%) among three distinct criteria for the metabolic syndrome in adolescents.

In face of such disparities, the validity of the metabolic syndrome diagnosis is presently being questioned. The same author who firstly defined it has discussed its clinical usefulness since the components are individually treated [39]. Another point of discussion is whether the components of the metabolic syndrome and the diagnosis always have the same importance in assessing the risk for developing cardiovascular diseases and whether the presence of two factors, and not three, would be sufficient to identify a patient at risk [40].

The existing criteria do not seem to be ideal for various reasons, such as: few longitudinal studies allowing for the evaluation of the metabolic syndrome's components from childhood to adulthood have been performed; family history concerning non-transmittable chronic diseases has not yet been thoroughly investigated; few studies have used fast insulinemia as an indicator of the glucose metabolism or cutoff points according to pubertal staging; the inclusion of BMI as an anthropometric indicator may overestimate the prevalence of the metabolic syndrome [40].

With such lack of agreement, some researchers have chosen to use multivariate analysis, for children, with the same components of the metabolic syndrome in adults [10]. Eisenmann (2008) [41] proposed the estimation of a score to diagnose the metabolic syndrome in children including the following components: abdominal circumference, triacylglycerols, HDL-C, blood pressure and glucose intolerance. The great difference in this method is the determination of the Z individual score for each child without dichotomization of variables, thus providing it with greater sensitivity. Recently, Eisenmann et al (2010) [42] analyzed the validity of a continuous metabolic syndrome (cMetS) score derived from principal component analysis based on the criteria by Cook et al (2003) [8]. cMetS score 3.72 was the optimal cutoff point with high sensitivity (100%) and high specificity (94%), and it meant the highest risk for the metabolic syndrome. However, this method has a major limitation: it is sample-specific.

The main limitation in this study was the absence of a control group consisting of eutrophic children, similarly to other studies [15,18,30,35]. The studies including a control group [11,20,30,34,37] showed no or low prevalence of the metabolic syndrome in eutrophic children. The prevalences of the metabolic syndrome may have been overestimated by the larger presence of obese children (56%) in relation to those overweight. The use of BMI as a component of the metabolic syndrome in the different criteria may also overestimate its prevalence, a condition described by another study [41].

The importance of this study in clinical practice for the decision concerning the criterion to be used to diagnose the syndrome and in the interpretation of results is noteworthy. Although prevalences differed according to the criterion adopted, agreement among the methods was considered to be satisfactory. The results in the present study can suggest that, in the same group of subjects, children with metabolic abnormalities will be diagnosed regardless of the diagnostic criteria selected.

It can also be speculated that the differences between the cutoff points of the components, namely, blood pressure, triacylglycerols, HDL-C and glycemia, are discreet. As previously discussed, the difference between the criteria is the choice for BMI or WC and fast glycemia or HOMA-IR and which reference to use in order to establish the diagnosis of alteration. Agreement on the use of the best anthropometric and glycidic indicator would reduce prevalence divergences among studies.

Additionally, it is recommended that the glucose, insulin and lipid levels of all children diagnosed with obesity should be periodically observed, and if required, immediate intervention should be made before the metabolic syndrome itself develops [21].

Besides of the metabolic syndrome diagnosis, other factors must be taken into account in cardiovascular risk evaluation in childhood. Dyslipidemia in childhood does not cause adverse health effects, such as acute myocardial infarction, but its long-term effects have been investigated. With the lack or scarcity of longitudinal studies on dyslipidemia in childhood and cardiovascular diseases in adulthood, studies make inferences, and a positive relation is found [43].

The latest recommendation from the American Association of Pediatrics for dyslipidemia screening is the presence of a family history of premature cardiovascular diseases or high total cholesterol concentrations, children whose families have an unknown family history or who have been diagnosed with overweight, obesity, high blood pressure and diabetes mellitus [44]. Several studies have shown a positive correlation between the presence of family history for obesity, systemic high blood pressure, dyslipidemia and type-2 diabetes mellitus and alteration in the metabolic-syndrome components [45-48].

Low weight at birth also influences the development of chronic diseases in adults, a phenomenon which is referred to as programming [49]. Growth rates in the first weeks of life are critical for later insulin resistance [50]. Fast weight gain soon after birth (0 to 6 months of life) has shown a direct relation with the presence of the metabolic syndrome components in adulthood [51].

The Non-alcoholic fatty liver disease is also related to metabolic disorders, and it is thought to be a result of obesity [52]. Kelishadi et al (2010) [53] have observed that alteration in hepatic enzymes increased with body weight gain, since it was twofold in overweight children and adolescents and fourfold in obese children and adolescents as compared to eutrophic individuals

Hence, an early diagnosis for overweight and the implementation of lifestyle changes are fundamental for preventing, controlling and managing obesity and its associated co-morbidities, such as the metabolic syndrome [54,55].

## Conclusions

Although some authors mentioned the difficulty to compare the prevalence of the metabolic syndrome, this study showed that the six criteria are correlated with each other. The prevalence of the metabolic syndrome varied from 10 to 16.5% and anthropometric indicators (BMI and WC) were the most altered components in all the criteria adopted. Additionally, the prevalence of the metabolic syndrome is higher in obese individuals as compared to those overweight. Therefore, in the same group of subjects, the prevalence of the metabolic syndrome is similar, regardless of the criterion selected.

## List of Abbreviations

BMI: body mass index; DBP: diastolic blood pressure; HOMA-IR: Homeostasis Model Assessment-Insulin Resistance; NCEP-ATPIII: National Cholesterol Education Program-Adult Treatment Painel III; SBP: systolic blood pressure; SSSF: subscapular skinfold; TSF: triceps skinfold; WC: waist circumference

## Competing interests

The authors declare that they have no competing interests.

## Authors' contributions

AEMR wrote the manuscript and collected the data. GDP corrected the manuscript and performed the statistical analysis. FM and GFCPG collected the data and corrected the manuscript. AFP corrected the manuscript. RCB read and approved the final version of the manuscript. All authors read and approved the final manuscript.
